# Controlling severe haemorrhage at myomectomy with mops: a case report

**DOI:** 10.1093/jscr/rjaf1014

**Published:** 2025-12-23

**Authors:** Nathan Chidiadi, Assumpta Nweke, Silas Nwali, Francis Okoroafor, Chuka Obi, Peter Nwokpoku, Moses Ikegbu, Justus Eze

**Affiliations:** Department of Obstetrics and Gynaecology, Alex Ekwueme Federal University Teaching Hospital, No. 1 Chidume Street, Behind State Prison Headquarters, P.M.B.102 Abakaliki, Ebonyi State, Nigeria; Department of Obstetrics and Gynaecology, Alex Ekwueme Federal University Teaching Hospital, No. 1 Chidume Street, Behind State Prison Headquarters, P.M.B.102 Abakaliki, Ebonyi State, Nigeria; Department of Obstetrics and Gynaecology, Alex Ekwueme Federal University Teaching Hospital, No. 1 Chidume Street, Behind State Prison Headquarters, P.M.B.102 Abakaliki, Ebonyi State, Nigeria; Department of Obstetrics and Gynaecology, Alex Ekwueme Federal University Teaching Hospital, No. 1 Chidume Street, Behind State Prison Headquarters, P.M.B.102 Abakaliki, Ebonyi State, Nigeria; Department of Obstetrics and Gynaecology, Alex Ekwueme Federal University Teaching Hospital, No. 1 Chidume Street, Behind State Prison Headquarters, P.M.B.102 Abakaliki, Ebonyi State, Nigeria; Department of Obstetrics and Gynaecology, Alex Ekwueme Federal University Teaching Hospital, No. 1 Chidume Street, Behind State Prison Headquarters, P.M.B.102 Abakaliki, Ebonyi State, Nigeria; Department of Obstetrics and Gynaecology, Alex Ekwueme Federal University Teaching Hospital, No. 1 Chidume Street, Behind State Prison Headquarters, P.M.B.102 Abakaliki, Ebonyi State, Nigeria; Department of Obstetrics and Gynaecology, Alex Ekwueme Federal University Teaching Hospital, No. 1 Chidume Street, Behind State Prison Headquarters, P.M.B.102 Abakaliki, Ebonyi State, Nigeria

**Keywords:** severe haemorrhage, myomectomy, transvaginal, intraabdominal

## Abstract

Interventions for controlling uterine bleeding during myomectomy, abound. When, however, the haemorrhage arises from low pressure bleeders in the retropelvic space and threatens patient’s exsanguination, hitherto unknown interventions become life-saving. The aim of this report is to bring to fore a case of profuse, unprecedented myomectomy-associated retropelvic haemorrhage that was successfully controlled with combined transvaginal and transabdominal pelvic mop packing. A multipara, who presented with a 2-year history of symptomatic uterine fibroids. Intra-operatively, she developed profuse haemorrhage after the excision of a submucous fundal fibroid in the retro-pelvic space. Two abdominal mops pushed trans-vaginally to pack the pelvis reduced the bleeding by 80%, and a transabdominal mop exerting pressure on the transvaginal mops stopped bleeding entirely. The mops were removed after 48 hours, and she recovered uneventfully. Deep pelvic haemorrhage complicating myomectomy may be successfully stopped by combined transvaginal and transabdominal pelvic packing to save life.

## Introduction

Leiomyomas, are the most common benign gynaecological tumour in premenopausal women [1]. Open myomectomy is the commonest conservative treatment of leiomyomas [2]. Myomectomy can be associated with life-threatening bleeding which may necessitate emergency blood transfusion [3]. Various methods have been used to reduce intraoperative blood loss during myomectomy. Neilson in his Cochrane review found significant reductions in blood loss with misoprostol weighted mean difference (WMD) of 149.00 ml, vasopressin and analogues (WMD –298.72 ml), bupivacaine plus epinephrine (WMD –68.60 ml) and pericervical tourniquet (WMD –1870.00 ml) [4]. There is limited evidence from a few RCTs that misoprostol, vasopressin, bupivacaine plus epinephrine, tourniquet in prevention of intraoperative blood loss [4]. Winata *et al.* documented that abdominal and pelvic packing can be used to stop recurrent bleeding during surgery. He documented that low-pressure veins and capillaries in the abdominal vault are compressed by the abdominal packing which is a mechanical, quick, efficient and cheap way of cessation of bleeding [5]. Kumar *et al.* in their case series involving intraabdominal pelvic packing for cytoreduction in case of ovarian carcinoma with diffuse oozing showed a significant reduction in blood loss [6].

Although many agents have been tried in the reduction of bleeding at myomectomy with some level of success, however, in cases of diffuse oozing, abdominal packing has been done with great success. Combined abdominal and vaginal packing can be of benefit in cases of diffuse oozing in the pelvis extending to the pouch of Douglas.

## Case report

A 41 year old para 3 trader with three living children, who presented to the gynaecology clinic with complaints of heavy menstrual flow and abdominal distension of 2 years. She used more than three heavily soaked pads in a day. The flow lasted for > 8 days each month, associated with lower abdominal pain and dizziness with no fainting attacks. She noticed abdominal mass about the same time which had progressively increased in size with associated loin pain. She attained menarche at 13 years and had a 4 day flow in a regular 28 days cycle.

On presentation, she was in no obvious distress, not febrile, not pale, not jaundiced, and not dehydrated and no pedal oedema. Her vital signs were normal. Abdominal examination revealed suprapubic distension with no areas of tenderness. There was a uterine mass of 30 weeks with multiple nodules; firm, non tender, and extending to the left loin area. Bimanual vaginal examination revealed a uterine mass of ~30 weeks extending into the cervix and obliterating the cervical canal. The adnexae and POD were free. A diagnosis of huge symptomatic uterine fibroid was made.

Laboratory findings showed a packed cell volume of 22% and transabdominal fibroids confirmed the presence of uterine fibroids. She had blood transfusion and was given haematinics. She was admitted in the proliferative phase of her next cycle, repeat packed cell volume was 36%.Consent was obtained and two units of blood were grouped and cross matched for the surgery. Intra-operatively, she developed profuse haemorrhage after the excision of a thick-stalked submucous fundal fibroid that grew into the retro-pelvic space and was adherent to the structures therein. Two abdominal mops pushed trans-vaginally to pack the pelvic reduced the bleeding by 80%, and a transabdominal mop exerting pressure on the transvaginal mops stopped bleeding entirely. The mops were removed after 48 hours, and she recovered uneventfully.

Abdominal drain was placed. The estimated blood loss was 3.5 L and had two units of blood transfused. She received antibiotics, haematinics, and analgesics. She had re-exploration after 48 hours and the mops were removed. She received another unit of blood post re-exploration packed cell volume was 28% and her post-operative period was uneventful.

## Discussion

**Figure 1:**
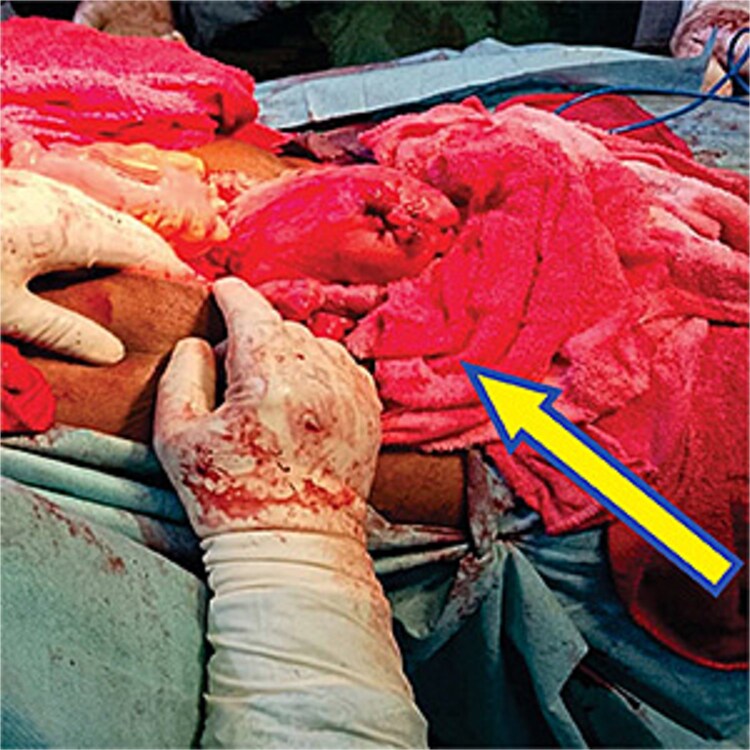
Intra abdominal mop insertion into the pelvis to control bleeding from multiple bleeders after insertion of intra vaginal mop.

**Figure 2:**
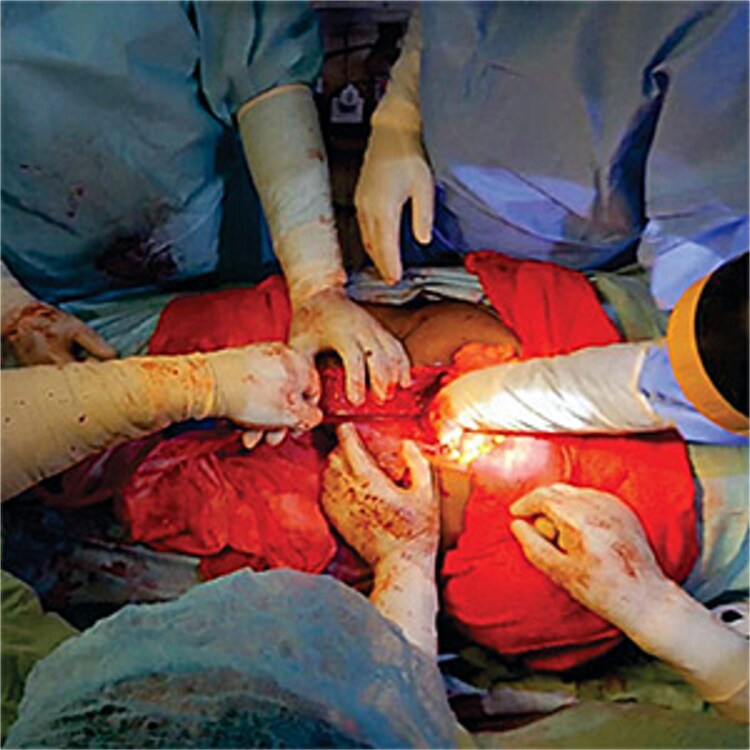
Observation for haemostasis and bleeding was noticed to have stopped.

Pelvic packing is a critical technique in the management of uncontrollable bleeding. A review of 11 cases by Bhosale *et al*. [7] in India reported that the pelvic pressure packing successfully controlled bleeding in 100% of cases without morbidity and mortality. Multiple bleeding sites especially in the pelvis made visualization of the bleeding points difficult. Also, attempts to place sutures were unsuccessful. Pelvic packing compresses the low-pressure veins and capillaries in the vaginal vault to decrease or stop the bleeding [8]. In this case, a combination of pelvic and vaginal packing stopped the bleeding. Figure 1 showed pelvis packed with abdominal mop to stop bleeding. The material commonly used for pelvic packing is mop the material used in this patient was abdominal mop as shown in Fig. 2. However, a study showed that a sterile bag filled with sterile gauze rolls was placed in the pelvic cavity but the infection rate was found to be high [8]. Other materials have been used but it is not readily available especially in the face of uncontrollable bleeding as seen in this case. Zhang *et al.* [9] in 15 trials with1136 patients, showed that pressure packing reduced the amount of postoperative transfusion, shortening the time of waiting and operating, and decreasing mortality due to uncontrolled hemorrhage when compared with endovascular intervention.

As already reported by Bhosale *et al.,* pelvic packing controlled bleeding as seen in this case but because of the oozing that persisted, a transvaginal pack maximally decreased bleeding as was in the index case and shown in Fig. 2.

## Conclusion

Haemorrhage in all cases is a source of worry to the patient and the managing physician. Simple, affordable, and effective methods of controlling bleeding should be employed to save the lives of our patients.

## References

[1] Florence AM, Fatehi M. Leiomyoma. [Updated 2023 Jul 17]. In: StatPearls [Internet]. Treasure Island (FL): StatPearls Publishing; 2025. Available from: https://www.ncbi.nlm.nih.gov/books/NBK538273/

[2] Conforti A, Mollo A, Ca A, et al. Techniques to reduce blood loss during open myomectomy: a qualitative review of literature. Eur J Obstetr Gynaecol Reproduct Biol 2015;192:90–5. 10.1016/j.ejogrb.2015.05.027 26189110

[3] Kongnyuy EJ, Wiysonge CS. Interventions to reduce haemorrhage during myomectomy for fibroids. Cochrane Database Syst Rev 2014;2014:CD005355. 10.1002/14651858.CD005355.pub5 17253552

[4] Neilson JP . Interventions to reduce haemorrhage during myomectomy for fibroids. Obstet Gynecol 2007;109:1197–8. 10.1097/01.AOG.0000263778.58572.25 17470606

[5] Winata IGS, Asmara AD. Abdominal packing for obstetric surgical uncontrollable hemorrhage. Eur J Med Health Sci 2022;4:70–3. 10.24018/ejmed.2022.4.4.1446

[6] Kumar S, Sharma JB, Karmakar D, et al. Combined intra-abdominal pelvic packing during cytoreductive surgery in advanced epithelial ovarian cancer: a case series. Arch Gynecol Obstet 2012;285:1125–32. 10.1007/s00404-011-2101-9 21984040

[7] Bhosale A, SKavya SH, Nandanwar YS, et al. Pelvic pressure packing for intractable obstetric and gynaecological hemorrhage in a tertiary care hospitaln. Int J Reproduct Contracept Obstet Gynecol 2018;7:4956–9. 10.18203/2320-1770.ijrcog20184947

[8] Naranjo-Gutiérrez LA, Oliva-Cristerna J, Ramírez-Montiel ML, et al. Pelvic packing with vaginal traction for the management of intractable hemorrhage. Int J Gynecol Obstet 2014;127:21–4. 10.1016/j.ijgo.2014.04.007 24950907

[9] Dong Z, Gong-zi Z, Ye P, et al. Pelvic packing or endovascular interventions: which should be given priority in managing hemodynamically unstable pelvic fractures? A systematic review and a meta analysis. Surg Open Sci 2024;19:146–57. 10.1016/j.sopen.2024.03.016 38721524 PMC11077165

